# Gout comorbidities: results from the Korean National Health and Nutrition Examination Survey

**DOI:** 10.1186/s42358-024-00413-8

**Published:** 2024-09-27

**Authors:** Hyemin Jeong, Young-Soo Chang, Chan Hong Jeon

**Affiliations:** 1 Division of Rheumatology, Department of Internal Medicine, Soonchunhyang University Bucheon Hospital, 170 Jomaru-ro, Bucheon, 14584 South Korea; 2 Department of Otorhinolaryngology-Head and Neck Surgery, Sanggye Paik Hospital, College of Medicine, Inje University, Seoul, South Korea

**Keywords:** Gout, Comorbidity

## Abstract

**Objectives:**

Gout is associated with several comorbidities. This study aimed to evaluate the prevalence of comorbidities in the Korean adult population with gout and investigated the association of gout with these comorbidities.

**Methods:**

Data from 15,935 (weighted *n* = 39,049,167) participants aged 19 years and older in the Korean National Health and Nutrition Examination Survey from 2019 to 2021 were used for analysis. Weighted prevalence and odds ratios (OR) of comorbidities in individuals with gout were compared to a non-gout population.

**Results:**

The weighted prevalence of gout was 2.1% (weighted *n* = 808,778). Among individuals with gout, 66.5% had metabolic syndrome, 54.9% had hypertension, 41.2% had hypercholesterolemia, 19.1% had diabetes, 13.5% had chronic kidney disease (CKD), 4.1% had myocardial infarction or angina, 3.8% had stroke, and 2.8% had rheumatoid arthritis (RA). After adjusting for socioeconomic and lifestyle characteristics, gout was independently associated with the increased prevalence of metabolic syndrome (male OR = 2.0, 95% confidence interval (CI): 1.5–2.8; female OR = 3.7, 95% CI: 1.5–9.2), hypercholesterolemia (male OR = 1.9, 95% CI: 1.4–2.5; female OR = 3.1, 95% CI: 1.3–7.5), CKD (male OR = 4.5, 95% CI: 2.7–7.3; female OR = 11.5, 95% CI: 4.1–32.1), and RA (male OR = 2.8, 95% CI: 1.1–7.1; female OR = 3.1, 95% CI: 1.1–8.7) compared to the non-gout population.

**Conclusions:**

Gout was associated with several comorbidities, including RA, in both males and females. These results suggest that the prevention and treatment of comorbidities at the individual level, carried out by clinicians, and knowledge of these comorbidities would help guide health policies for the Korean population.

## Introduction

Gout is the most common type of inflammatory arthritis in adults [1]. Gout is characterized as an arthritic condition that occurs due to the accumulation of monosodium urate crystals in the joints, resulting from the prolonged elevation of uric acid levels [2]. Various factors, including dietary and lifestyle choices, medications, and kidney function, can influence the levels of uric acid in the bloodstream [3]. Apart from the significant pain it causes, gout is also linked to a variety of comorbidities, including cardiovascular disease, kidney disease, metabolic syndrome, and diabetes [4, 5]. In certain instances, such as chronic kidney disease (CKD), it is evident that the coexisting condition plays a role in advancing gout [6]. Conversely, gout or hyperuricemia alone plays a part in the development of comorbidities associated with gout [7]. Population-based epidemiologic studies found that the prevalence and incidence of gout is increasing worldwide [8–10]. As the prevalence of gout increases, gout and comorbidities accompanying gout can have a significant adverse impact on public health. Therefore, understanding the patterns of comorbid conditions in gout is crucial.

According to the National Health and Nutrition Examination Survey 2007–2008 in the United States, gout was associated with an increased prevalence of hypertension, CKD, obesity, diabetes, nephrolithiasis, myocardial infarction, heart failure, and stroke compared to those without gout [11]. The multicenter study disclosed an association between gout and metabolic syndrome, highlighting obesity and dyslipidemia as the primary factors linked to this syndrome in the study participants [12]. The prevalence of metabolic syndrome in patients with gout was approximately 50% in a tertiary hospital study [13]. However, few studies have been conducted on comorbidities in Korean patients with gout using large-scale data.

This study aimed to evaluate the prevalence of comorbidities in the Korean adult population with gout and investigated the association of gout with these comorbidities using data from the Korean National Health and Nutrition Examination Survey (KNHANES).

## Methods

This cross-sectional study utilized comorbidity and laboratory data collected during the 2019–2021 KNHANES. This nationwide survey, conducted periodically by the Korea Centers for Disease Control and Prevention, aims to assess the health and nutritional status of the Korean population [14].

### Study population

Participants were selected using the proportional allocation and systematic sampling method with multistage stratification to create a representative Korean population [15]. Although individual participants do not typically represent the entire Korean population, this survey provides representative estimates of the non-institutionalized Korean civilian population by utilizing sample weights. These weights were adjusted to address factors such as discrepancies in household and population counts between the time of sample design and the survey, unequal sampling rates, and non-response errors in the survey. This adjustment was made to enhance the representativeness and precision of health-related behaviors, the prevalence of chronic diseases, and associated estimations for the target population, which was the Korean population.

A total of 22,559 participants in the 2019–2021 KNHANES dataset were initially considered. Among them, a group of 18,691 individuals aged 19 and above was chosen for the study. The analysis excluded 2,756 participants due to missing data related to gout diagnosis or demographic variables. Finally, a total of 15,935 individuals (weighted *n* = 39,049,167) were included for analysis. This study received approval from the Institutional Review Board of Soonchunhyang University Hospital (IRB No. 2023-04-002).

### Data collection

Standardized interviews were conducted individually by a professional investigator using a well-established questionnaire that encompassed demographic and socioeconomic characteristics to gather data on demographic variables.

#### Assessment of gout

Gout was defined in the questionnaire as “Gout diagnosed by a physician (yes/no)” through a standardized interview. The specific question asked was, “Has a physician diagnosed you with gout?”

#### Assessment of comorbidities

The KNHANES dataset encompasses a wealth of information on the association between gout and various diseases across diverse categories, including well-studied conditions like cardiovascular disease and kidney disorders, as well as relatively understudied ones such as allergic conditions, rheumatoid arthritis, cancers, and depression. Recognizing this breadth, our analysis extended beyond comorbidities traditionally associated with gout, such as cardiovascular and kidney diseases, to encompass a wide range of conditions included in the KNHANES survey. We aimed to explore the association between gout and a comprehensive array of diseases, beyond the commonly investigated ones.

The following comorbidities were defined in the same way as the gout diagnosis. Information was collected on comorbidities, including stroke, myocardial infarction or angina, osteoarthritis, rheumatoid arthritis (RA), osteoporosis, pulmonary tuberculosis, asthma, thyroid disease, thyroid cancer, depression, atopic dermatitis, allergic rhinitis, sinusitis, otitis media, cataract, and chronic hepatitis B that were diagnosed by a physician. Hypertension was defined as having a systolic blood pressure of 140 mmHg or higher or a diastolic blood pressure of 90 mmHg or higher. Individuals taking antihypertensive medication were also categorized within this group. Diabetes was defined as having a fasting blood glucose level of 126 mg/dL or higher, receiving a diagnosis of diabetes from a physician, using hypoglycemic drugs or insulin injections, or having an HbA1c level of 6.5% or higher. Hypercholesterolemia was defined as having a total cholesterol level greater than 240 mg/dL or using cholesterol-lowering drugs. Metabolic syndrome was defined according to the modified National Cholesterol Education Program Adult Treatment Panel III (ATP III) definition [16]. Current ATP III criteria were used to define metabolic syndrome if any three of the following five traits were present: (1) central obesity (waist circumference ≥ 90 cm in Asian men or ≥ 80 cm in Asian women), (2) hypertriglyceridemia (a fasting serum triglyceride level ≥ 150 mg/dL or drug treatment for elevated triglycerides), (3) decreased high-density lipoprotein (HDL) cholesterol levels (serum HDL cholesterol <40 mg/dL in men or <50 mg/dL in women or drug treatment for low HDL cholesterol), (4) elevated blood pressure (BP) (systolic blood pressure ≥ 130 mmHg and/or diastolic blood pressure ≥ 85 mmHg or drug treatment for elevated BP), and (5) hyperglycemia (fasting plasma glucose levels ≥ 100 mg/dL or drug treatment for elevated blood glucose). CKD was defined as an estimated glomerular filtration rate (eGFR) of < 60. The eGFR was calculated using the Modification of Diet in Renal Disease (MDRD) study equation: eGFR (mL/min/1.73 m²) = 175 − (serum creatinine) − 1.154 − (age) − 0.203 − (0.742 if female) [17]. Blood samples were collected in the morning after a minimum 8-hour fasting period. The samples were promptly refrigerated and transported in cold storage to the central testing facility.

#### Assessment of confounders

Socioeconomic and lifestyle characteristics were assessed as potential confounders in the relationship between gout and comorbidities. Socioeconomic factors, such as age, income, education, and marital status, were considered, alongside lifestyle characteristics including alcohol consumption, smoking habits, and body mass index (BMI). These information were collected through the KNHANES questionnaire. Heavy alcohol drinking was defined as consuming alcohol more than two times per week in the year preceding the interview.

### Statistical analysis

Descriptive statistics were used to identify the characteristics of the study population. Clinical comparisons were performed using *t*-tests, and the Mann–Whitney U-test was performed for continuous variables, as appropriate. Chi-square tests were used for categorical variables. Logistic regression analysis for each sex individually was performed to investigate the association between gout and comorbidities. Firstly, the crude analysis, without adjustment, was carried out, and then the multivariate analysis was done, adjusting for socioeconomic factors (age, income, education, marital status) and lifestyle characteristics (alcohol consumption, smoking, and BMI).

Odds ratios (ORs) with 95% confidence intervals (CIs) were calculated for each comorbidity. P-values were corrected by Bonferroni’s method for multiple testing. Statistical analyses were performed using SAS (SAS version 9.4; SAS Institute, Cary, NC, USA). All *P*-values were two-sided, and *P <* 0.05 was considered statistically significant.

## Results

### Demographic and frequency of comorbidities in individuals with gout

The average age of the total study population was 47.5 years, with 50.2% being male. The weighted prevalence of gout was determined to be 2.1% (weighted *n* = 808,778). Among participants with gout, 66.5% had metabolic syndrome, 54.9% had hypertension, 41.2% had hypercholesterolemia, 19.1% had diabetes, 17.2% had cataracts, 14.7% had allergic rhinitis, 13.5% had CKD, 9.3% had osteoarthritis, 5.7% had pulmonary tuberculosis, 4.1% had myocardial infarction or angina, 3.8% had stroke, and 2.8% had RA.

Demographic and clinical characteristics based on the presence or absence of gout for both males and females are presented in Table 1. The mean age of both males and females with gout was higher than that of the non-gout population. In males, alcohol consumption was more frequent in the gout population than in the non-gout population, while in females, there was no significance in alcohol consumption between those with and without gout. The prevalence of comorbidities, including hypertension, diabetes, hypercholesterolemia, metabolic syndrome, CKD, osteoarthritis, rheumatoid arthritis, and cataracts, was higher in both males and females with gout compared to the individuals without gout. The prevalence of stroke was higher in males with gout compared to those without gout, whereas in the female group, there was no significant difference.

**Table 1:** Demographic and clinical characteristics according to the presence or absence of gout, stratified by sex

Variable	Total (*n* = 15,935, weighted *n* = 39,049,167)	Male (*n* = 7125, weighted *n* = 19,582,378)			Female (*n* = 8810, weighted *n* = 19,466,789)		
		Non-gout (*n* = 6853, weighted *n* = 18,848,463)	Gout (*n* = 272, weighted *n* = 733,915)	*p*	Non-gout (*n* = 8769, weighted *n* = 19,391,926)	Gout (*n* = 41, weighted *n* = 74,863)	*p*
Age	47.5 ± 0.24	46.4 ± 0.3	52.6 ± 0.8	<0.001	48.4 ± 0.3	59.0 ± 3.2	<0.001
Sex							
Male	19,582,377 (50.2)	-	-		-	-	
Female	19,466,789 (49.8)	-	-		-	-	
Income				0.733			0.329
Low	9,342,721 (23.9)	4,506,857 (23.9)	178,588 (24.3)		4,640,033 (23.9)	17,242 (23.0)	
Mid-low	9,703,107 (24.9)	4,733,258 (25.1)	163,145 (22.3)		4,784,949 (24.7)	21,755 (29.1)	
Mid-high	10,057,075 (25.7)	4,827,461 (25.6)	209,208 (28.5)		4,994,301 (25.8)	26,106 (34.9)	
High	9,946,264 (25.5)	4,780,887 (25.4)	182,974 (24.9)		4,972,643 (25.6)	9760 (13.0)	
Education				0.131			<0.001
Elementary school	4,512,872 (11.6)	1,396,889 (7.4)	64,556 (8.8)		3,016,408 (15.6)	35,019 (46.8)	
Middle school	3,005,375 (7.7)	1,313,535 (7.0)	70,840 (9.7)		1,616,891 (8.3)	4,109 (5.5)	
High school	14,524,844 (37.2)	7,335,105 (38.9)	307,068 (41.8)		6,860,435 (35.4)	22,235 (29.7)	
College education	17,006,076 (43.5)	8,802,934 (46.7)	291,450 (39.7)		7,898,192 (40.7)	13,500 (18.0)	
Marital status				<0.001			
Married	29,115,056 (74.6)	13,071,815 (69.4)	622,832 (84.9)		15,348,008 (79.2)	72,401 (96.7)	0.002
Unmarried	9,934,110 (25.4)	5,776,648 (30.6)	111,083 (15.1)		4,043,918 (20.8)	2462 (3.3)	
Smoking status				<0.001			<0.001
Non-smoker	22,096,248 (56.6)	5,072,743 (26.9)	123,029 (16.7)		16,851,390 (86.9)	49,086 (65.6)	
Ex-smoker	9,391,017 (24.0)	7,567,034 (40.2)	407,151 (55.5)		1,413,031 (7.3)	3801 (5.1)	
Current smoker	7,561,902 (19.4)	6,208,686 (32.9)	203,735 (27.8)		1,127,505 (5.8)	21,976 (29.3)	
Alcohol consumption				0.005			0.118
Never	9,720,958 (24.9)	3,061,711 (16.2)	139,259 (19.0)		6,484,105 (33.4)	35,883 (47.9)	
1 ≤ week	20,918,986 (53.5)	9,877,313 (52.4)	301,085 (41.0)		10,712,579 (55.2)	28,008 (37.4)	
2–3 / week	6,011,531 (15.4)	4,043,588 (21.5)	183,662 (25.0)		1,777,921 (9.2)	6361 (8.5)	
4 ≥ week	2,397,691 (6.2)	1,865,851 (9.9)	109,908 (15.0)		417,321 (2.2)	4611 (6.2)	
Heavy alcohol drinking*	8,409,223 (21.5)	5,909,439 (31.4)	293,570 (40.0)	0.010	2,195,242 (11.3)	10,972 (14.7)	0.558
Body mass index, kg/m²	24.2 ± 0.1	24.8 ± 0.1	25.9 ± 0.3	<0.001	23.4 ± 0.1	25.0 ± 0.8	0.055
BMI, kg/m²				0.004			0.016
<18.5	1,590,965 (4.1)	447,897 (2.4)	14,133 (1.9)		1,122,510 (5.8)	6426 (8.6)	
18.5–22.9	14,210,041 (36.5)	5,137,892 (27.3)	121,695 (16.6)		8,934,482 (46.2)	15,972 (21.3)	
23–24.9	8,826,329 (22.6)	4,928,946 (26.1)	190,079 (25.9)		3,692,826 (19.1)	14,477 (19.4)	
≥25	14,358,007 (36.8)	8,333,727 (44.2)	408,008 (55.6)		5,578,283 (28.9)	37,989 (50.7)	
Uric acid		6.03 ± 0.02	7.03 ± 0.11	<0.001	4.50 ± 0.01	5.27 ± 0.27	0.005
Hypertension	10,617,356 (27.2)	5,462,241 (29.0)	406,958 (55.4)	<0.001	4,710,826 (24.3)	37,332 (49.9)	0.006
Diabetes	4,857,238 (12.7)	2,651,816 (14.5)	129,501 (18.4)	<0.001	2,056,239 (10.9)	19,683 (26.7)	0.010
Hypercholesterolemia	8,990,039 (23.6)	388,1477 (21.2)	275,945 (39.2)	<0.001	4,787,761 (25.2)	44,857 (60.9)	<0.001
Metabolic syndrome	15,152,398 (38.8)	7,838,515 (41.6)	479,241 (65.3)	<0.001	6,776,266 (34.9)	58,376 (78.0)	<0.001
Stroke	658,359 (1.7)	360,895 (1.9)	29,426 (4.0)	0.017	267,126 (1.4)	912 (1.2)	0.902
Myocardial infarction or stroke	912,023 (2.3)	520,228 (2.8)	32,824 (4.5)	0.125	358,283 (1.9)	687 (0.9)	0.479
Chronic kidney disease	1,089,237 (2.8)	521,005 (2.8)	88,349 (12.0)		458,798 (2.4)	21,085 (28.2)	<0.001
Osteoarthritis	3,299,182 (8.5)	695,872 (3.7)	54,632 (7.4)	0.001	2,528,373 (13.0)	20,304 (27.1)	0.010
Rheumatoid arthritis	562,947 (1.4)	133,340 (0.7)	15,769 (2.2)	0.008	407,224 (2.1)	6614 (8.8)	0.003
Osteoporosis	2,150,204 (5.5)	151,882 (0.8)	5513 (0.8)	0.8859	1,974,457 (10.2)	18,351 (24.5)	0.003
Pulmonary tuberculosis	1,232,082 (3.2)	696,626 (3.7)	45,026 (6.1)	0.049	489,540 (2.5)	889 (1.2)	0.440
Asthma	1,229,542 (3.2)	552,088 (2.9)	3508 (0.5)	<0.001	671,471 (3.5)	2475 (3.3)	0.952
Thyroid disease	1,480,968 (3.8)	256,451 (1.4)	7021 (1.0)	0.612	1,208,003 (6.2)	9493 (12.7)	0.138
Thyroid cancer	452,155 (1.2)	88,082 (0.5)	5165 (0.7)	0.622	358,221 (1.9)	687 (0.9)	0.480
Depression	1,808,462 (4.6)	524,798 (2.8)	23,953 (3.3)	0.567	1,247,826 (6.4)	11,885 (15.9)	0.029
Atopic dermatitis	1,611,705 (4.1)	769,114 (4.1)	7177 (1.0)	0.004	831,782 (4.3)	3632 (4.9)	0.875
Allergic rhinitis	6,564,167 (16.8)	2,709,130 (14.4)	109,011 (14.9)	0.853	3,736,482 (19.3)	9544 (12.8)	0.243
Sinusitis	2,786,699 (7.1)	1,367,504 (7.3)	41,330 (5.6)	0.435	1,371,128 (7.1)	6737 (9.0)	0.631
Cataract	2,401,192 (6.2)	1,576,860 (8.4)	115,683 (15.8)	<0.001	2,612,699 (13.5)	23,625 (31.6)	0.002
Chronic hepatitis B	4,328,867 (11.1)	309,657 (1.6)	10,839 (1.5)	0.875	148,549 (0.8)	2607 (3.5)	0.024

*BMI* body mass index, *SBP* systolic blood pressure, *DBP* diastolic blood pressure, *HDL* high-density lipoprotein, *LDL* low-density lipoprotein, *AST* aspartate transferase, *ALT* alanine transaminase, *BUN* blood urea nitrogen, *eGFR* estimated glomerular filtration rate

*Heavy alcohol drinking, defined as more than two times per week

### Logistic regression analyses for comorbidities based on the presence of gout

Univariable and multivariable logistic regression analyses for comorbidities based on the presence of gout in both males and females are presented in Table 2. After adjusting for socioeconomic and lifestyle characteristics, gout was found to be independently associated with an increased prevalence of metabolic syndrome (male OR = 2.0, 95% CI: 1.5–2.8; female OR = 3.7, 95% CI: 1.5–9.2), hypercholesterolemia (male OR = 1.9, 95% CI: 1.4–2.5; female OR = 3.1, 95% CI: 1.3–7.5), CKD (male OR = 4.5, 95% CI: 2.7–7.3; female OR = 11.5, 95% CI: 4.1–32.1), and RA (male OR = 2.8, 95% CI: 1.1–7.1; female OR = 3.1, 95% CI: 1.1–8.7) compared to the non-gout population. The prevalence of hypertension was significantly associated with gout in males, whereas no significant association was observed in females (male OR = 2.4, 95% CI: 1.7–3.3; female OR = 1.4, 95% CI: 0.7–2.7).

**Table 2:** ORs (95% CI) for comorbidities among participants with gout compared to non-gout participants

Variable	Male				Female			
	Unadjusted OR	*p*	Adjusted OR*	*p*	Unadjusted OR	*p*	Adjusted OR*	*p*
Hypertension	3.05 (2.29, 4.05)	<0.001	2.35 (1.67, 3.30)	<0.001	3.1 (1.55, 6.22)	0.002	1.43 (0.77, 2.66)	0.261
Diabetes mellitus	1.33 (0.92, 1.93)	0.129	0.95 (0.63, 1.45)	0.821	3.00 (1.40, 6.43)	0.005	1.47 (0.67, 3.22)	0.336
Hypercholesterolemia	2.40 (1.80, 3.19)	<0.001	1.88 (1.4, 2.54)	<0.001	4.62 (2.31, 9.24)	<0.001	3.10 (1.27, 7.52)	0.013
Metabolic syndrome	2.64 (1.97, 3.54)	<0.001	2.01 (1.46, 2.78)	<0.001	6.61 (2.84, 15.38)	<0.001	3.72 (1.50, 9.23)	0.005
Stroke	2.14 (1.13, 4.07)	0.020	1.60 (0.82, 3.12)	0.169	0.88 (0.12, 6.43)	0.902	0.38 (0.05, 3.07)	0.363
Myocardial infarction or stroke	1.65 (0.86, 3.15)	0.130	1.2 (0.61, 2.35)	0.605	0.49 (0.07, 3.65)	0.489	0.25 (0.03, 1.98)	0.189
Chronic kidney disease	4.81 (3.21, 7.21)	<0.001	4.45 (2.71, 7.31)	<0.001	16.18 (7.50, 34.90)	<0.001	11.52 (4.13, 32.11)	<0.001
Osteoarthritis	2.1 (1.32, 3.34)	0.002	1.62 (0.99, 2.63)	0.053	2.48 (1.22, 5.07)	0.013	1.16 (0.56, 2.42)	0.690
Rheumatoid arthritis	3.08 (1.29, 7.36)	0.012	2.81 (1.12, 7.06)	0.029	4.52 (1.54, 13.24)	0.006	3.05 (1.06, 8.73)	0.039
Osteoporosis	0.93 (0.35, 2.45)	0.886	0.77 (0.29, 2.08)	0.612	2.87 (1.37, 6.00)	0.005	1.71 (0.82, 3.55)	0.151
Pulmonary tuberculosis	1.70 (1.00, 2.92)	0.053	1.52 (0.87, 2.66)	0.140	0.46 (0.06, 3.43)	0.452	0.38 (0.05, 2.81)	0.343
Asthma	0.16 (0.05, 0.51)	0.002	0.16 (0.05, 0.52)	0.002	0.95 (0.20, 4.57)	0.953	0.64 (0.13, 3.11)	0.577
Thyroid disease	0.70 (0.18, 2.80)	0.615	0.56 (0.14, 2.27)	0.417	2.19 (0.76, 6.31)	0.148	2.05 (0.72, 5.86)	0.179
Thyroid cancer	1.51 (0.29, 7.85)	0.625	1.24 (0.25, 6.3)	0.791	0.49 (0.07, 3.66)	0.489	0.62 (0.08, 4.82)	0.646
Depression	1.18 (0.67, 2.07)	0.568	1.21 (0.68, 2.16)	0.513	2.75 (1.07, 7.04)	0.036	1.9 (0.71, 5.1)	0.201
Atopic dermatitis	0.23 (0.08, 0.69)	0.009	0.38 (0.12, 1.14)	0.084	1.14 (0.23, 5.71)	0.876	3.07 (0.62, 15.18)	0.169
Allergic rhinitis	1.04 (0.69, 1.56)	0.851	1.26 (0.84, 1.91)	0.269	0.61 (0.27, 1.41)	0.250	0.84 (0.30, 2.34)	0.744
Sinusitis	0.76 (0.39, 1.51)	0.437	0.87 (0.44, 1.72)	0.686	1.3 (0.45, 3.8)	0.631	1.55 (0.5, 4.8)	0.452
Cataract	2.05 (1.42, 2.95)	0.0001	1.66 (1.05, 2.63)	0.031	2.96 (1.43, 6.14)	0.004	1.53 (0.65, 3.56)	0.328
Hepatitis B	0.90 (0.23, 3.47)	0.876	0.82 (0.21, 3.21)	0.780	4.68 (1.07, 20.42)	0.041	3.07 (0.72, 13.11)	0.130

*Adjusted for age, income, education, smoking, alcohol, body mass index, and marriage

## Discussion

We evaluated the comorbidities of patients with gout using the nationwide KNHANES conducted by the Korean government. Frequently associated comorbidities in patients with gout included metabolic syndrome, hypertension, hypercholesterolemia, diabetes, CKD, and RA, listed in descending order. Following adjustment for socioeconomic and lifestyle factors, gout demonstrated an independent association with a metabolic syndrome, hypercholesterolemia, CKD, and RA in males and females compared to individuals without gout. Additionally, the presence of gout was significantly linked to hypertension in male participants.

In nationwide study in Taiwan, hypercholesterolemia (OR 1.63), hypertension (OR 2.00), renal insufficiency (OR 2.06) were the common comorbidities of gout [18]. Hyperlipidemia (59.7%) and hypertension (53.7%) were common comorbidities of gout in Japan [19]. Hypertension and obesity are common comorbidities in Western countries. In USA, frequently associated comorbidities in gout was hypertension (74%), chronic kidney disease (71%), obesity (53%), and diabetes (26%) [11]. In UK, obesity (27.7%), hypertension (17.5%), myocardial infarction (7.4%), and heart failure (7.1%) [20]. In Germany, diabetes (25.9%) was most common comorbidities in gout, followed by hypertension (18.5%), and heart failure (10.5%) [20].

Metabolic syndrome is increased among gout patients compared to the general population [12, 13, 21]. We previously reported that hyperuricemia was associated with an increased prevalence of metabolic syndrome in both males and females [22]. Hyperuricemia was also associated with the number of metabolic syndrome components [22]. Several studies demonstrated a relationship between elevated uric acid levels and insulin resistance [23, 24]. As serum insulin diminishes the renal excretion of uric acid, uric acid levels could increase as a result of hyperinsulinemia in metabolic syndrome [25].

Hypertension is associated with a 2 to 3 folds increase the risk of gout [26, 27]. Meta-analysis of 25 studies showed that hyperuricemia can modestly increase the risk of hypertension with a dose-dependent relationship [28]. Although there is a clear relationship between gout and hypertension, there remains insufficient evidence elucidating how gout precipitates the hypertension. Several studies might provide evidence for uric acid-induced hypertension [29]. Uric acid directly simulates the vascular smooth muscle proliferation and induces renal arteriolopathy, leading to hypertension in experimental rat model [30]. Serum uric acid was strongly associated with the risk allele (T) of the rs734553 polymorphism of the glucose transporter 9 gene, and individuals with risk allele showed higher systolic blood pressure than control group [31]. Allopurinol use was independently associated with fall in both systolic and diastolic blood pressure after allopurinol initiation [32].

The association between gout and hypercholesterolemia was not fully understood. Previous studies shown that hypercholesterolemia is associated with increased risk of gout [33–35]. In nationwide, population-based cohort study in Taiwan, overall incidence risk of hyperlipidemia in gout patients was 2.55 fold compared to that in non-gout population [36]. Higher baseline serum uric acid levels and increased serum uric acid levels over 5 years is an independent risk factor for developing high LDL cholesterol [37]. Serum uric acid might play a part in promoting lipogenesis while inhibiting fatty acid oxidation [38, 39]. Wu et al. reported that uric acid lowering therapy decreased serum cholesterol levels in patients with gout who did not receive lipid lowering therapy [40]. Uric acid lowering medications are proposed to have an effect on decreasing the expression of lipogenesis-related genes [36].

Chronic kidney disease is common in patients with gout. A meta-analysis of epidemiological studies reported that gout was associated with both CKD stage ≥ 3 (OR = 2.41, 95% CI: 1.86–3.11) and nephrolithiasis (OR = 1.77, 95% CI: 1.43–2.19) [41]. A diminished GFR poses a risk of premature tophi emergence, indicating that renal function could influence the severity of gout [42]. Conversely, the prevalence of gout is elevated in individuals diagnosed with CKD [43]. Kidney impairment may arise due to comorbid conditions, such as hypertension, diabetes, and the use of nonsteroidal anti-inflammatory drugs [44]. Hyperuricemia-mediated endothelial dysfunction and renovascular disease also cause renal impairment [45]. A previous meta-analysis study suggested that CKD patients with uric acid-lowering therapy tended to show superior eGFR preservation compared to patients without uric acid-lowering therapy [46]. Appropriately managing uric acid levels in patients with gout is speculated to contribute to the prevention of CKD [47].

The prevalence of RA was significantly higher in participants with gout compared to the non-gout population (2.8% vs. 1.4%, *p* = 0.033). Gout was independently associated with an increased prevalence of RA (male OR = 2.8, 95% CI: 1.1–7.1; female OR = 3.1, 95% CI: 1.1–8.7) compared to the non-gout population after adjusting for socioeconomic and lifestyle characteristics. In contrast, the prevalence of osteoarthritis was not significantly different between the gout and non-gout populations. In the literature, gout and RA occur in the same patients. However, it seems that the incidence is rare. To date, approximately 60 cases of concurrent RA and gout have been documented in the literature [48]. Jebakumar et al. reported that among 813 patients with RA, 22 developed gout, and the 25-year cumulative incidence of gout in patients with RA was 2.4%. Gout development was more common among patients diagnosed with RA in recent years (1995–2007) compared to those diagnosed in earlier years (1980–1994) [49]. In this study, older age, male sex, and obesity were risk factors for gout in patients with RA [49]. A single-center retrospective study showed that 13 patients met both classification criteria for RA and gout over a period of 13 years. All of the 13 patients had at least one comorbidity, such as hypertension, CKD, or obesity [48]. To the best of our knowledge, no analysis of the prevalence of RA in patients with gout has been performed. The reasons for the higher prevalence of RA in patients with gout compared to those without gout, as observed in the current study, are not definitively clear. Gout and RA are inflammatory arthritis conditions with different clinical and laboratory features. Despite their differences, RA and gout share clinical features, such as polyarticular involvement and subcutaneous nodules. Studies have suggested a potential close connection between uric acid and inflammatory responses. Elevated serum uric acid levels show a positive correlation with tumor necrosis factor (TNF)-α, interleukin (IL)-6, and C-reactive protein (CRP) [50, 51]. Wang et al. reported that serum uric acid levels were significantly higher in patients with RA than in the healthy controls. Furthermore, rheumatoid factor (RF) and anti-cyclic citrullinated peptide (anti-CCP) were positively correlated with serum uric acid levels in patients with RA [52]. Additional studies incorporating more epidemiological data are needed to understand the relationship between gout and RA further.

When examining comorbidities among gout patients based on the presence of hyperuricemia (male, uric acid ≥ 7 mg/dL; female, uric acid ≥ 6 mg/dL), the prevalence of metabolic syndrome was higher in the hyperuricemia group in both males and females. However, there was no significant difference in multivariable analysis among gout-afflicted males with or without hyperuricemia (data were not shown). The limited number of female gout patients precluded multivariable analysis due to the small sample size. Furthermore, since uric acid was measured only once, there may have been limitations in determining whether uric acid control was achieved.

This study had several limitations. First, the determination of gout and RA relied on participant-provided information during interviews, lacking the application of specific classification criteria for these conditions. Second, an analysis was conducted to examine the association between gout and malignancy, but no significant correlation with gout was found. Due to the limited number of malignancy cases in both males and females, multivariable analysis was often unfeasible. Therefore, results were not provided for most malignancies, excluding thyroid cancer. Third, the cross-sectional design of this study precluded the establishment of causality. Despite the limitations, the current study provides comprehensive cross-sectional data on gout and comorbidities in Korean adult populations.

## Conclusions

Gout was independently associated with several comorbidities, including metabolic syndrome, hypercholesterolemia, CKD, and RA in both males and females. These findings indicate the importance of healthcare professionals paying attention to underlying comorbidities in individuals with gout.

## Data Availability

The datasets used and/or analyzed during the current study available from the corresponding author on reasonable request.

## References

[1] Singh JA, Gaffo A. Gout epidemiology and comorbidities. Semin Arthritis Rheum. 2020;50(3):S11–6. 32620196 10.1016/j.semarthrit.2020.04.008

[2] Shi Y, Mucsi AD, Ng G. Monosodium urate crystals in inflammation and immunity. Immunol Rev. 2010;233(1):203–17. 10.1111/j.0105-2896.2009.00851.x 20193001

[3] Gliozzi M, Malara N, Muscoli S, Mollace V. Treat Hyperuricemia. Int J Cardiol. 2016;213:23–7. 10.1016/j.ijcard.2015.08.087 26320372

[4] Bardin T, Richette P. Impact of comorbidities on gout and hyperuricaemia: an update on prevalence and treatment options. BMC Med. 2017;15(1):123. 28669352 10.1186/s12916-017-0890-9 PMC5494879

[5] Singh JA, Gaffo A. Gout epidemiology and comorbidities. Semin Arthritis Rheum. 2020;50(3S):S11–6. 32620196 10.1016/j.semarthrit.2020.04.008

[6] Roughley MJ, Belcher J, Mallen CD, Roddy E. Gout and risk of chronic kidney disease and nephrolithiasis: meta-analysis of observational studies. Arthritis Res Ther. 2015;17:1–12. 10.1186/s13075-015-0610-9 PMC4404569 25889144

[7] Karis E, Crittenden DB, Pillinger MH. Hyperuricemia, gout, and related comorbidities: cause and effect on a two-way street. South Med J. 2014;107(4):235–41. 24937517 10.1097/SMJ.0000000000000082

[8] Rai SK, Aviña-Zubieta JA, McCormick N, De Vera MA, Shojania K, Sayre EC, et al. The rising prevalence and incidence of gout in British Columbia, Canada: population-based trends from 2000 to 2012. Semin Arthritis Rheum. 2017;46(4):451–6. 28040245 10.1016/j.semarthrit.2016.08.006 PMC5315679

[9] Trifirò G, Morabito P, Cavagna L, Ferrajolo C, Pecchioli S, Simonetti M, et al. Epidemiology of gout and hyperuricaemia in Italy during the years 2005–2009: a nationwide population-based study. Ann Rheum Dis. 2013;72(5):694–700. 10.1136/annrheumdis-2011-201254 22736095

[10] Kim J-W, Kwak SG, Lee H, Kim S-K, Choe J-Y, Park S-H. Prevalence and incidence of gout in Korea: data from the national health claims database 2007–2015. Rheumatol Int. 2017;37(9):1499–506. 28676911 10.1007/s00296-017-3768-4

[11] Zhu Y, Pandya BJ, Choi HK. Comorbidities of gout and hyperuricemia in the US general population: NHANES 2007–2008. Am J Med. 2012;125(7):679–e871. 22626509 10.1016/j.amjmed.2011.09.033

[12] Rho YH, Choi SJ, Lee YH, Ji JD, Choi KM, Baik SH, et al. The prevalence of metabolic syndrome in patients with gout: a multicenter study. J Korean Med Sci. 2005;20(6):1029–33. 16361817 10.3346/jkms.2005.20.6.1029 PMC2779304

[13] Jung JH, Song GG, Ji JD, Lee YH, Kim JH, Seo YH, et al. Metabolic syndrome: prevalence and risk factors in Korean gout patients. Korean J Intern Med. 2018;33(4):815–22. 27729624 10.3904/kjim.2016.062 PMC6030414

[14] Oh K, Kim Y, Kweon S, Kim S, Yun S, Park S, et al. Korea National Health and Nutrition Examination Survey, 20th anniversary: accomplishments and future directions. Epidemiol Health. 2021;43:e2021025. 33872484 10.4178/epih.e2021025 PMC8289475

[15] Kweon S, Kim Y, Jang MJ, Kim Y, Kim K, Choi S, et al. Data resource profile: the Korea National Health and Nutrition Examination Survey (KNHANES). Int J Epidemiol. 2014;43(1):69–77. 24585853 10.1093/ije/dyt228 PMC3937975

[16] Grundy SM, Cleeman JI, Daniels SR, Donato KA, Eckel RH, Franklin BA, et al. Diagnosis and management of the metabolic syndrome: an American Heart Association/National Heart, Lung, and Blood Institute Scientific Statement. Circulation. 2005;112(17):2735–52. 16157765 10.1161/CIRCULATIONAHA.105.169404

[17] Lamb EJ, Tomson CRV, Roderick PJ. Estimating kidney function in adults using formulae. Ann Clin Biochem. 2005;42(5):321–45. 16168188 10.1258/0004563054889936

[18] Tu F-Y, Lin G-T, Lee S-S, Tung Y-C, Tu H-P, Chiang H-C. Prevalence of gout with comorbidity aggregations in southern Taiwan. Joint Bone Spine. 2015;82(1):45–51. 25238950 10.1016/j.jbspin.2014.07.002

[19] Koto R, Nakajima A, Horiuchi H, Yamanaka H. Real-world treatment of gout and asymptomatic hyperuricemia: a cross-sectional study of Japanese health insurance claims data. Mod Rheumatol. 2021;31(1):261–9. 32552370 10.1080/14397595.2020.1784556

[20] Annemans L, Spaepen E, Gaskin M, Bonnemaire M, Malier V, Gilbert T, et al. Gout in the UK and Germany: prevalence, comorbidities and management in general practice 2000–2005. Ann Rheum Dis. 2008;67(7):960–6. 17981913 10.1136/ard.2007.076232 PMC2564789

[21] Choi HK, Ford ES, Li C, Curhan G. Prevalence of the metabolic syndrome in patients with gout. Third Natl Health Nutr Examination Surv. 2007;57(1):109–15. 10.1002/art.22466 17266099

[22] Jeong H, Moon JE, Jeon CH. Hyperuricemia is Associated with an increased prevalence of metabolic syndrome in a General Population and a decreased prevalence of diabetes in men. J Rheumatic Dis. 2020;27(4):247–60.

[23] Lee J, Sparrow D, Vokonas PS, Landsberg L, Weiss ST. Uric acid and coronary heart disease risk: evidence for a role of uric acid in the obesity-insulin resistance syndrome. The normative aging study. Am J Epidemiol. 1995;142(3):288–94. 7631632 10.1093/oxfordjournals.aje.a117634

[24] Rathmann W, Funkhouser E, Dyer AR, Roseman JM. Relations of hyperuricemia with the various components of the insulin resistance syndrome in young black and white adults: the CARDIA study. Coronary artery Risk Development in Young adults. Ann Epidemiol. 1998;8(4):250–61. 9590604 10.1016/s1047-2797(97)00204-4

[25] Quiñones Galvan A, Natali A, Baldi S, Frascerra S, Sanna G, Ciociaro D, et al. Effect of insulin on uric acid excretion in humans. Am J Physiol. 1995;268(1 Pt 1):E1–5. 7840165 10.1152/ajpendo.1995.268.1.E1

[26] Roubenoff R, Klag MJ, Mead LA, Liang K-Y, Seidler AJ, Hochberg MC. Incidence and risk factors for gout in White men. JAMA. 1991;266(21):3004–7. 1820473

[27] Choi HK, Atkinson K, Karlson EW, Curhan G. Obesity, weight change, hypertension, diuretic use, and risk of gout in men: the Health professionals follow-up study. Arch Intern Med. 2005;165(7):742–8. 15824292 10.1001/archinte.165.7.742

[28] Wang J, Qin T, Chen J, Li Y, Wang L, Huang H, et al. Hyperuricemia and risk of incident hypertension: a systematic review and meta-analysis of observational studies. PLoS ONE. 2014;9(12):e114259. 25437867 10.1371/journal.pone.0114259 PMC4250178

[29] Seong-Kyu K. Interrelationship of uric acid, gout, and metabolic syndrome: Focus on Hypertension, Cardiovascular Disease, and insulin resistance. J Rheumatic Dis. 2018;25(1):19–27.

[30] Mazzali M, Kanellis J, Han L, Feng L, Xia YY, Chen Q, et al. Hyperuricemia induces a primary renal arteriolopathy in rats by a blood pressure-independent mechanism. Am J Physiol Ren Physiol. 2002;282(6):F991–7. 10.1152/ajprenal.00283.2001 11997315

[31] Mallamaci F, Testa A, Leonardis D, Tripepi R, Pisano A, Spoto B, et al. A polymorphism in the major gene regulating serum uric acid associates with clinic SBP and the white-coat effect in a family-based study. J Hypertens. 2014;32(8):1621–8; discussion 8. 24805955 10.1097/HJH.0000000000000224

[32] Beattie CJ, Fulton RL, Higgins P, Padmanabhan S, McCallum L, Walters MR, et al. Allopurinol initiation and change in blood pressure in older adults with hypertension. Hypertension. 2014;64(5):1102–7. 25135183 10.1161/HYPERTENSIONAHA.114.03953

[33] Chen S-Y, Chen C-L, Shen M-L, Kamatani N. Trends in the manifestations of gout in Taiwan. Rheumatology. 2003;42(12):1529–33. 13130146 10.1093/rheumatology/keg422

[34] Qiu L, Cheng X-Q, Wu J, Liu J-T, Xu T, Ding H-T, et al. Prevalence of hyperuricemia and its related risk factors in healthy adults from Northern and northeastern Chinese provinces. BMC Public Health. 2013;13(1):664. 23866159 10.1186/1471-2458-13-664 PMC3722003

[35] Dehlin M, Jacobsson L, Roddy E. Global epidemiology of gout: prevalence, incidence, treatment patterns and risk factors. Nat Rev Rheumatol. 2020;16(7):380–90. 32541923 10.1038/s41584-020-0441-1

[36] Fang Y-J, Wu T-Y, Lin C-L, Su C-Y, Li J-R, Chung Y-L, et al. Effects of Urate-lowering therapy on risk of Hyperlipidemia in gout by a Population-based Cohort Study and on *in vitro* hepatic lipogenesis-related gene expression. Mediat Inflamm. 2020;2020:8890300. 10.1155/2020/8890300 PMC7683152 33273891

[37] Kuwabara M, Borghi C, Cicero AFG, Hisatome I, Niwa K, Ohno M, et al. Elevated serum uric acid increases risks for developing high LDL cholesterol and hypertriglyceridemia: a five-year cohort study in Japan. Int J Cardiol. 2018;261:183–8. 29551256 10.1016/j.ijcard.2018.03.045

[38] Lanaspa MA, Sanchez-Lozada LG, Choi Y-J, Cicerchi C, Kanbay M, Roncal-Jimenez CA, et al. Uric acid induces hepatic steatosis by generation of mitochondrial oxidative stress: potential role in fructose-dependent and-independent fatty liver. J Biol Chem. 2012;287(48):40732–44. 10.1074/jbc.M112.399899 PMC3504786 23035112

[39] Choi Y-J, Shin H-S, Choi HS, Park J-W, Jo I, Oh E-S, et al. Uric acid induces fat accumulation via generation of endoplasmic reticulum stress and SREBP-1c activation in hepatocytes. Lab Invest. 2014;94(10):1114–25. 10.1038/labinvest.2014.98 25111690

[40] Wu J, Zhang Y-P, Qu Y, Jie L-G, Deng J-X, Yu Q-H. Efficacy of uric acid-lowering therapy on hypercholesterolemia and hypertriglyceridemia in gouty patients. Int J Rheum Dis. 2019;22(8):1445–51. 10.1111/1756-185X.13652 31317680

[41] Roughley MJ, Belcher J, Mallen CD, Roddy E. Gout and risk of chronic kidney disease and nephrolithiasis: meta-analysis of observational studies. Arthritis Res Therapy. 2015;17(1):90. 10.1186/s13075-015-0610-9 PMC4404569 25889144

[42] Dalbeth N, House ME, Horne A, Taylor WJ. Reduced creatinine clearance is associated with early development of subcutaneous tophi in people with gout. BMC Musculoskelet Disord. 2013;14:363. 24359261 10.1186/1471-2474-14-363 PMC3878111

[43] Krishnan E. Reduced glomerular function and prevalence of gout: NHANES 2009-10. PLoS ONE. 2012;7(11):e50046. 23209642 10.1371/journal.pone.0050046 PMC3507834

[44] Kazancioğlu R. Risk factors for chronic kidney disease: an update. Kidney Int Supplements. 2013;3(4):368–71. 10.1038/kisup.2013.79 PMC4089662 25019021

[45] Jin M, Yang F, Yang I, Yin Y, Luo JJ, Wang H, et al. Uric acid, hyperuricemia and vascular diseases. Front Biosci (Landmark Ed). 2012;17:656. 10.2741/3950 PMC3247913 22201767

[46] Maruyama Y, Kumagai T, Sugano N, Yoshida S, Ichida K, Uchida S. Effect of uric acid-lowering therapy on renal function in patients with chronic kidney disease: a systematic review and meta-analysis. Ren Replace Therapy. 2021;7(1):44.

[47] Kannuthurai V, Gaffo A. Management of patients with gout and kidney disease: a review of available therapies and common missteps. Kidney360. 2023;4(9):e1332–40. 37526648 10.34067/KID.0000000000000221 PMC10550007

[48] Olaru L, Soong L, Dhillon S, Yacyshyn E. Coexistent rheumatoid arthritis and gout: a case series and review of the literature. Clin Rheumatol. 2017;36(12):2835–8. 29022182 10.1007/s10067-017-3856-6

[49] Jebakumar AJ, Udayakumar PD, Crowson CS, Matteson EL. Occurrence of gout in rheumatoid arthritis: it does happen! A population-based study. Int J Clin Rheumtol. 2013;8(4):433–7. 24443656 10.2217/ijr.13.45 PMC3891477

[50] Shi Y, Mucsi AD, Ng G. Monosodium urate crystals in inflammation and immunity. Immunol Rev. 2010;233(1):203–17. 20193001 10.1111/j.0105-2896.2009.00851.x

[51] Spiga R, Marini MA, Mancuso E, Di Fatta C, Fuoco A, Perticone F, et al. Uric acid is Associated with inflammatory biomarkers and induces inflammation Via activating the NF-κB signaling pathway in HepG2 cells. Arterioscler Thromb Vasc Biol. 2017;37(6):1241–9. 28408375 10.1161/ATVBAHA.117.309128

[52] Wang Z, Wang W, Xiang T, Gong B, Xie J. Serum uric acid as a diagnostic biomarker for Rheumatoid Arthritis-Associated interstitial lung disease. Inflammation. 2022;45(4):1800–14. 35314903 10.1007/s10753-022-01661-w PMC9197871

